# Retrospective study of the diagnostic utility of Spec fPLin the assessment of 274 sick cats

**DOI:** 10.1111/jvim.15797

**Published:** 2020-05-26

**Authors:** Cherrie Lee, Aarti Kathrani, Jill Maddison

**Affiliations:** ^1^ The Royal Veterinary College Hatfield United Kingdom

**Keywords:** feline, pancreatitis, pancreatic lipase

## Abstract

**Background:**

Serum feline pancreatic lipase immunoreactivity (fPL) commonly is used in the assessment of sick cats suspected to have pancreatitis but its diagnostic utility is debated.

**Objectives:**

To evaluate the diagnostic utility of the Spec fPL test and selected serum biochemistry tests in the diagnosis of pancreatitis in cats.

**Animals:**

Two hundred seventy‐four client‐owned cats presented to a university teaching hospital in the United Kingdom, from April 2013 to May 2017, in which Spec fPL was measured.

**Methods:**

Cats were classified into 1 of 4 groups based on clinical signs (all cats), ultrasonographic findings (all cats) and histopathological or cytological assessment of the pancreas where available (9 cats) regardless of Spec fPL concentration. The groups were (a) definite pancreatitis (n = 9), (b) probable pancreatitis (n = 49), (c) possible pancreatitis (n = 139), and (d) unlikely pancreatitis (n = 77). Spec fPL and selected serum biochemistry test results were compared among groups.

**Results:**

Serum fPL concentrations >5.3 μg/L were classified as positive and concentrations <3.5 μg/L were classified as negative. There was a significantly (*P* = .03) lower proportion of false‐positive results (cats unlikely to have pancreatitis, n = 77, with a positive fPL, n = 8, 10%) than false‐negative results (cats with definite or probable pancreatitis, n = 58, with a negative fPL result, n = 14, 24%). None of the selected biochemical tests were helpful diagnostically.

**Conclusion and Clinical Importance:**

A positive Spec fPL result indicates that pancreatitis is a probable diagnosis, but the test cannot be used to rule the diagnosis out.

**Abbreviations**
ALP: alkaline phosphatase
ALT: alanine aminotransferase
fPL: feline pancreatic lipase

## 1 INTRODUCTION

Pancreatitis is relatively common in cats. However, reaching a definitive diagnosis is challenging because of the nonspecific clinical signs,^1^, ^2^ the variable sensitivity and specificity of the diagnostic tests available,^3^ and the challenge of finding a gold standard diagnostic test against which other diagnostic tests can be assessed. Nonspecific clinical signs of pancreatitis in cats include anorexia, lethargy, vomiting, diarrhea, dehydration, abdominal pain, abdominal distension, weight loss, and pyrexia.^1^, ^3^, ^4^

Diagnosis of pancreatitis in cats usually involves a combination of clinical suspicion, evaluation of clinical pathology test results, ultrasonographic evidence of pancreatitis, and measurement of serum feline pancreatic lipase immunoreactivity (fPL).^1^, ^5^ Because no gold standard diagnostic test is available, clinicians must assess test results critically in the context of the clinical presentation.^6^

Lipase is secreted by several tissues and hence measuring the total serum activity of this enzyme is of no diagnostic value in the diagnosis of pancreatitis in cats.^2^ Pancreatic lipase, however, is exclusively secreted by the pancreas.^6^, ^7^ This was demonstrated in dogs, because dogs with exocrine pancreatic insufficiency had no canine pancreatic lipase immunoreactivity (cPL) in their serum.^8^, ^9^ However, no similar study has been reported in cats.^7^ Regardless, the amino acid sequence of feline pancreatic lipase should be different from that of lipase secreted by other tissues, which in turn should generate a specific immunologic response.^9^ The IDEXX laboratories developed the Spec fPL, a quantitative ELISA in 2008 for feline PL, as they had for the canine Spec cPL.^6^

Clinical pathology results reported in cats with pancreatitis include hyperbilirubinemia, hypocalcemia, and hypoalbuminemia, but there are conflicting reports on whether or not clinically relevant differences in these variables occur in cats with pancreatitis.^3^, ^6^

Our aims were to evaluate the diagnostic utility of the Spec fPL test and selected biochemical tests in the diagnosis of pancreatitis in cats presented to a small animal referral teaching hospital in the United Kingdom. Because no gold standard to assess the diagnostic performance of Spec fPL is available,^2^ a combination of diagnostic findings (clinical signs and ultrasonography in all cats, histopathology and cytology in some cats) was used to reach a diagnosis of definite, probable, possible, or unlikely pancreatitis.

## 2 MATERIALS AND METHODS

### 2.1 Animals

The Spec fPL results for 300 client‐owned cats admitted to a referral teaching hospital in the United Kingdom between April 2014 and May 2017 were obtained from the records of the hospital's veterinary diagnostic laboratory. Inclusion criteria for the study were all cats in which Spec fPL was measured, for which signalment and description of clinical signs at the time of blood sampling were recorded, and that had ultrasonographic assessment of the pancreas performed by a board‐certified specialist or supervised resident in diagnostic imaging in the teaching hospital. Twenty‐six cats that did not have an abdominal ultrasound examination performed were excluded.

The study was approved by the Social Science Research Ethical Review Board at the teaching hospital (URN number: SR2017‐1073).

### 2.2 Recording and data collection

The clinical records for all cats were reviewed and the following variables associated with the specific visit at which fPL was measured were recorded: signalment (age, breed, sex, and neuter status), presenting clinical signs, selected biochemistry test results (alanine aminotransferase [ALT] and alkaline phosphatase [ALP] activities, serum albumin, total calcium and bilirubin concentrations), and abdominal ultrasound findings, focusing on the pancreas, biliary system, and peritoneum.

Keywords that reflect the ultrasonographic changes reported for cats in the definite and probable pancreatitis groups were hypoechoic, enlargement, thickened, irregular margins, and hyperechoic peripancreatic fat.^10^

If available, the appearance of the pancreas at exploratory laparotomy, pancreatic cytology or histopathology, and the findings at necropsy were recorded.

Keywords that reflected the visual changes seen in cats in the definite pancreatitis group were firm, nodular, congested, duct distension, inflamed, and edematous. Keywords that reflected the histopathological or cytological changes seen in the definite pancreatitis group were lymphocytic or neutrophilic inflammation or both, necrosis, edema, and hemorrhage.

### 2.3 Classification of cases

Each case was categorized into 1 of 4 groups: (a) definite pancreatitis, (b) probable pancreatitis, (c) possible pancreatitis, and (d) unlikely pancreatitis regardless of the concentration of the Spec fPL according to the criteria described below (Table 1).

**TABLE 1:** Characteristics used to classify feline cases into definite pancreatitis, probable pancreatitis, possible pancreatitis, and unlikely pancreatitis

Definite pancreatitis All clinical signs and clinical pathology could be explained by pancreatitis Ultrasonographic changes consistent with pancreatitis Cytologic or histopathologic changes on pancreatic biopsy and/or visible changes in the pancreas on exploratory laparotomy or necropsy examination	Probable pancreatitis All clinical signs and clinical pathology could be explained by pancreatitis Ultrasonographic changes consistent with pancreatitis No visual, cytological, or histological examination of the pancreas performed
Possible pancreatitis Most clinical signs and history could be explained by pancreatitis No ultrasonographic evidence for pancreatitis No visual, cytological, or histological examination of the pancreas performed	Unlikely pancreatitis Clinical signs and history could be explained by pancreatitis but there was presence of another disease that could explain the presenting signs, for example, hypertrophic cardiomyopathy (HCM), renal disease, and gastrointestinal neoplasia No ultrasonographic evidence for pancreatitis No visual, cytological, or histological examination of the pancreas performed

In cats categorized as definite pancreatitis, all clinical signs and clinical pathology test results could be explained by pancreatitis, and no other identified concurrent disease was present that could explain the clinical signs. They had ultrasonographic changes consistent with pancreatitis and had cytological or histopathologic changes in pancreatic biopsy specimens, visible changes in the pancreas at exploratory laparotomy or necropsy examination or both.

In cats categorized as probable pancreatitis, all clinical signs and clinical pathology test results could be explained by pancreatitis and no other identified concurrent disease was present that could explain the clinical signs. They had ultrasonographic changes consistent with pancreatitis. No visual, cytological, or histological examination of the pancreas was performed.

In cats categorized as possible pancreatitis, most clinical signs and history could be explained by pancreatitis. However, they had no ultrasonographic evidence for pancreatitis and no visual, cytological, or histological examination of the pancreas was performed.

For cats categorized as unlikely pancreatitis, the clinical signs and history could be explained by pancreatitis but another disease was present that could explain the presenting signs (eg, hypertrophic cardiomyopathy, renal disease, gastrointestinal neoplasia). No ultrasonographic evidence for pancreatitis was present and no visual, cytological, or histological examination of the pancreas was performed.

### 2.4 Statistical analysis

The Spec fPL concentrations were not normally distributed (D'Agostino and Pearson normality test) and therefore nonparametric statistical analysis was performed. Median Spec fPL concentrations for each group were compared using the Kruskal‐Wallis test followed by Dunns multiple comparisons test if a significant difference was detected. Chi‐squared analysis was used to compare the proportion of positive (>5.3 μg/L) and negative (<3.6 μ/L) fPL results in each group.

Serum albumin, total bilirubin and calcium concentrations and ALT and ALP activities were analyzed among groups by using the Kruskal‐Wallis test followed by Dunns multiple comparisons test if a significant difference between groups was detected. The relationship between Spec fPL and bilirubin concentrations was assessed by using Pearson correlation.

Statistical tests were performed by using the GraphPad Prism Version 7.00. Significance was defined as *P* < .05. Comparison of proportions test was performed by using the MedCalc statistical software, which uses the “N‐1” Chi‐squared test.

## 3 RESULTS

### 3.1 Diagnostic classification

Of the 274 cats included in the study, 3.3% (n = 9) were assessed as having definite pancreatitis, 17.9% (n = 49) as probable pancreatitis, 50.7% (n = 139) as possible pancreatitis, and 28.1% (n = 77) as unlikely pancreatitis. The inclusion criteria used to classify the cats diagnostically are described in detail in the Methods section and in Table 1.

### 3.2 Study population

Sixty‐one percent of the cats were neutered males (n = 167) and all cats assessed as definite pancreatitis were neutered males (n = 9). Thirty‐seven percent (n = 101) were neutered females with the remaining cats being intact males (n = 2) or intact females (n = 4). The age of the cats ranged from 4 months to 19 years (median 2 years) with no significant difference in median age among the 4 groups (*P* = .6). The most commonly presented breeds included domestic shorthair (56.7%, n = 156), domestic longhair (6.9%, n = 19), Bengal (4.7%, n = 12), Burmese (4.7%, n = 13), and British shorthair (4.7%, n = 13). Other breeds included Birman, Siamese, Maine Coon, and Persian. There were >3 times as many Bengal cats in the definite (11.1%) and probable (10.2%) groups compared to the possible (2.9%) and unlikely (2.6%) groups. Burmese cats also were overrepresented in the probable (10.2%) group compared with the possible (3.6%) and the unlikely (3.9%) groups. No Burmese cat was found in the definite group.

### 3.3 Clinical signs

The most common clinical signs reported in all groups were lethargy, anorexia, vomiting, and weight loss (Table 2). Other clinical signs noted in all groups were polyuria and polydipsia, abdominal pain, and pyrexia.

**TABLE 2:** The 4 most common clinical signs presented by each of the 3 groups of cats, n = number of cats

	Combined definite and probable pancreatitis (n = 58)	Possible pancreatitis (n = 139)	Unlikely pancreatitis (n = 77)
1.	Lethargy (75.8%)	Anorexia (74.3%)	Anorexia (62.3%)
2.	Anorexia (66.1%)	Lethargy (61%)	Weight loss (54.5%)
3.	Vomiting (53.2%)	Vomiting (51.5%)	Lethargy (50.6%)
4.	Weight loss (50%)	Weight loss (44.9%)	Vomiting (33.8%)

### 3.4 Feline pancreatic lipase immunoreactivity (fPL) concentrations

**FIGURE 1:**
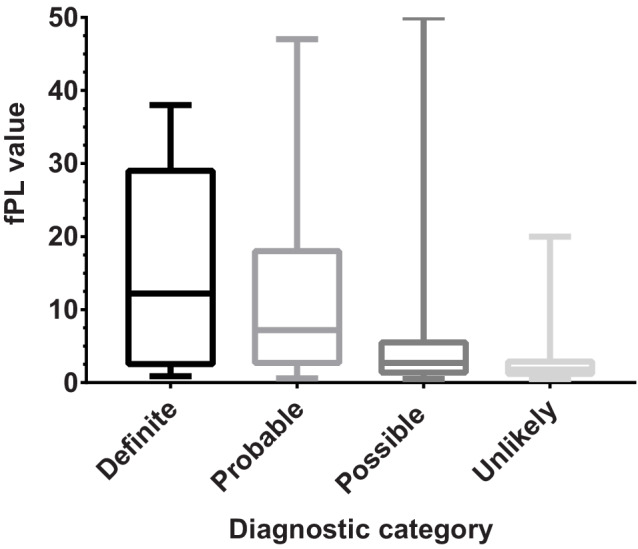
Box and Whiskers plot (median and range) comparing Spec fPL concentrations in each of the 4 groups of cats (definite pancreatitis n = 9, probable pancreatitis n = 49, possible pancreatitis n = 139, unlikely pancreatitis n = 77)

Spec fPL concentrations in the 274 cats ranged from 0.5 to 50 μg/L with a median of 2.6 μg/L (Table 3). The median fPL for the definite group was 12.2 μg/L (range, 0.9‐38 μg/L), the median for the probable group was 7.2 μg/L (range, 0.6‐47 μg/L), the median for the possible group was 2.7 μg/L (range, 0.5‐50 μg/L), and the median for the unlikely group was 1.8 μg/L (range, 0.5‐20 μg/L). A significant difference in fPL concentration was found between groups (*P* < .0001), with the following groups showing significant differences in concentrations after multiple comparison tests: definite versus unlikely (*P* = .007), probable versus possible (*P* = .0001), probable versus unlikely (*P* < .0001), and possible versus unlikely (*P* = .03; Figure 1). Because no significant difference was found between the fPL results for cats classified as definite versus probable pancreatitis, the results were combined for further analysis.

**TABLE 3:** Median, mean, and range of Spec fPL concentrations for each of the 4 groups of cats, n = number of cats

Group	Median Spec fPL (μg/L)	Mean Spec fPL (μg/L)	Range Spec fPL (μg/L)
Definite pancreatitis (n = 9)	12.2	14.7	0.9‐38
Probable pancreatitis (n = 49)	7.2	11.9	0.6‐47
Possible pancreatitis (n = 139)	2.7	5.8	0.5‐50
Unlikely pancreatitis (n = 77)	1.8	2.8	0.5‐20

**FIGURE 2:**
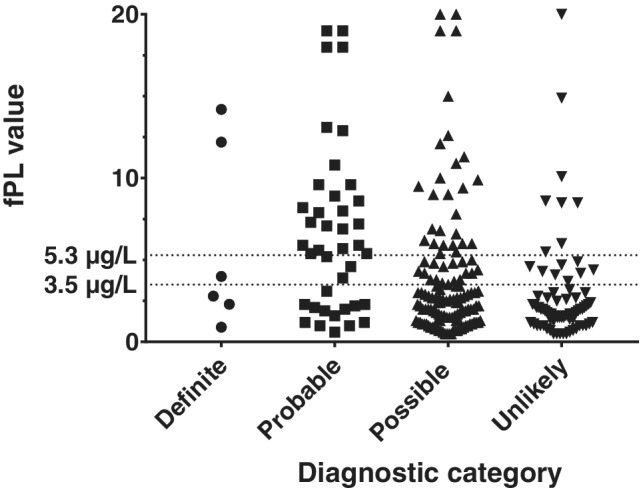
Scatter plot showing the distribution of Spec fPL concentrations up to 20 μg/L in each of the 4 groups of cats with reference range cutoff values of positive >5.3 μg/L and negative <3.6 μg/L. Twenty‐one data points are omitted as they were greater than 20 μg/L (3 definite pancreatitis, 9 probable pancreatitis, and 9 possible pancreatitis)

The median and range of fPL concentrations for each group are illustrated in Figure 1. The cutoff for positive, equivocal, and negative results was based on the reference range for the fPL test (0.7‐3.5 μg/L). Above 5.3 μg/L is considered consistent with pancreatitis and a result >5.3 μg/L was defined as a positive result. Results between 3.6 and 5.3 μg/L were considered intermediate and were defined as equivocal. Results ≤3.5 μg/L were defined as negative. The distribution of fPL results up to 20 μg/L is shown in Figure 2 to more clearly illustrate the distribution of results in each group <3.5 μg/L (negative results) and >5.3 μg/L (positive results). The number and proportion of cats in each fPL category in each group are shown in Table 4.

**TABLE 4:** Proportion of cats with positive (>5.3 μg/L), equivocal (3.5‐5.3 μg/L), and negative (<3.5 μg/L) fPL results for each of the groups of cats, n = number of cats

Diagnosis	Positive % (n) >5.3 μg/L	Equivocal % (n) 3.5‐5.3 μg/L	Negative % (n) <3.5 μg/L
Definite (n = 9)	55.5% (5)	11.1% (1)	33.3% (3)
Probable (n = 49)	67.3% (33)	10.2% (5)	22.4% (11)
Definite and probable (n = 58)	65.5% (38)	10.3% (6)	24.1% (14)
Possible (n = 139)	25.9% (36)	14.4% (20)	59.7% (83)
Unlikely (n = 77)	10.4% (8)	10.4% (8)	79.2% (61)

Chi‐squared analysis of the combined definite plus probable, possible, and unlikely groups showed that a significant difference was present in the proportion of cats with positive (>5.3 μg/L) and negative (<3.6 μg/L) fPL results in the groups (*P* < .0001). There was a significantly (*P* = .03) lower proportion of false‐positive results (cats with a positive fPL result of >5.3 μg/L classified as unlikely to have pancreatitis, 10%; n = 8) compared with false‐negative results (cats with definite or probable pancreatitis with a negative fPL result of <3.5 μg/L, 24; n = 14).

### 3.5 Serum biochemistry

No significant differences were found between combined definite and probable pancreatitis versus possible and unlikely pancreatitis groups for serum albumin (*P* = .4) and total ALT concentrations (*P* = .1) and ALP (*P* = .5) and calcium (*P* = .25) activities (Table 5).

**TABLE 5:** Select biochemistry parameters (median values) in the combined definite and probable pancreatitis, possible pancreatitis, and unlikely pancreatitis groups, n = number of cats

Test	Reference range	Combined definite and probable pancreatitis (n = 58)	Possible pancreatitis (n = 139)	Unlikely pancreatitis (n = 77)	*P* value
Albumin	28‐42 g/L	31.3	30.5	30.5	.37
ALT	25‐130 U/L	60	54	49	.10
ALP	11‐58 U/L	34.5	22	23	.05
Calcium	2.07‐2.8 mmol/L	2.28	2.29	2.25	.25

The overall median total bilirubin concentration was 2.2 μmol/L (reference range, 0‐15 μmol/L). A significant difference in total bilirubin concentration was found between the combined definite and probable pancreatitis group and the possible pancreatitis group (median combined definite and probable pancreatitis groups, 1.9 μmol/L; possible pancreatitis group, 2.5 μmol/L; *P* = .0005) and between the possible pancreatitis group and the unlikely pancreatitis group (median unlikely pancreatitis group = 1.9 μmol/L; *P* < .0001). No significant difference was found between the combined definite and probable group and the unlikely group (*P* = 1.00). The mean, median and range for the combined definite and probable, possible, and unlikely pancreatitis groups are shown in Table 6.

**TABLE 6:** The median, mean, and range of total bilirubin concentration in the combined definite and probable pancreatitis, possible pancreatitis, and unlikely pancreatitis groups, n = number of cats

Groups	Total bilirubin concentration (μmol/L)			
	Median	Mean	Range	Comparison between groups
Combined definite and probable pancreatitis (n = 58)	2.5	15.5	0‐194.7	Significantly different from possible pancreatitis (*P* = .0005)
Possible pancreatitis (n = 139)	2.5	17.1	0‐179.2	Significantly different from combined define and probable pancreatitis (*P* = .0005) and unlikely pancreatitis (*P* < .0001)
Unlikely pancreatitis (n = 77)	1.9	4.4	0‐132.6	Significantly different from possible pancreatitis (*P* < .0001)

No correlation was found between Spec fPL concentrations and bilirubin concentrations when concentrations from all of the groups were compared (*r* = 0.118, *P* = .06) nor when the results from the combined definite pancreatitis and probable pancreatitis groups were assessed (*r* = 0.01, *P* = .9).

## 4 DISCUSSION

The diagnosis of pancreatitis in cats remains difficult because of a lack of a gold standard test against which diagnostic modalities can be compared.^6^ Many authors have suggested that a combination of diagnostic tests is needed to reach a diagnosis.^3^, ^5^, ^11^ We retrospectively evaluated the diagnostic utility of the Spec fPL test by using a multiangle diagnostic approach in 274 cats.

The comparison of positive and negative results in the different groups of cats in our study showed that the proportion of probable false positives (ie, cats unlikely to have pancreatitis but with fPL > 5.3 μg/L) was low at 10%. This frequency may be even lower, because it was not possible in this group of cats to unequivocally rule out the presence of pancreatitis at the time of their final diagnosis. The proportion of false negatives (the proportion of cats with clinical signs and ultrasonographic changes consistent with pancreatitis but fPL <3.5 μg/L) was significantly higher at 24%. Therefore, in our population of cats, fPL had good diagnostic utility for ruling in the diagnosis (albeit with some false positives) but lower diagnostic utility in ruling out the diagnosis, because of the higher proportion of false‐negative results. The cats in our study were assessed at a university teaching hospital. Therefore, the false‐positive and false‐negative rates observed in our study may not apply to other populations in which the prevalence of pancreatitis is higher or lower.

It was not possible to calculate the sensitivity and specificity of the fPL test in our study because of the lack of a gold standard test performed in all cats that would allow the diagnosis to be definitively ruled in or out. Although histopathological assessment of the pancreas has been considered the most sensitive diagnostic tool compared to other diagnostic modalities,^2^ it can only be utilized to calculate the sensitivity and specificity of the fPL test if every cat in the study is biopsied. Histopathological assessment also presents challenges.

In our study, the small number of cats in which the pancreas was inspected visually or sampled reflects the realities of clinical practice where pancreatic biopsy is a seldom utilized diagnostic tool because of its invasive nature and cost. Pancreatic biopsy or cytology can be contraindicated in severe acute pancreatitis because of the invasiveness of the procedure.^12^ In addition, a surgeon suitably skilled to perform the biopsy may not be available, obtaining client consent may be difficult, and the progression of disease or presence of other concurrent diseases could considerably limit the diagnostic value of histopathology.^2^, ^3^ Moreover, pancreatic lesions can be focal and therefore normal histopathology result does not necessarily rule out the diagnosis.^12^ In addition, a tissue sample with microscopic pancreatic changes does not always correlate with the presence of clinical disease.^12^ There is also a lack of standardized histopathological grading for pancreatitis in cats.^5^, ^13^ In addition, histopathologic evidence of pancreatitis has been found in 45% of apparently healthy cats,^4^ which presents challenges in interpreting the clinical relevance of pancreatic inflammation, especially if mild. Thus, in practice, after consideration of the advantages and disadvantages of pancreatic biopsy, and often the need for immediate treatment of the patient, most cases, as in our study, do not usually involve this diagnostic procedure.^14^

Regardless, sensitivity and specificity of fPL have been reported from necropsy studies. One study used histopathological assessment in 60 cats presented for necropsy as a reference standard.^15^ The sensitivity and specificity of the Spec fPL test in that study (with 5.3 μg/L as upper reference range limit) ranged from 42.1 to 61.1% and 69.0 to 100%, respectively, depending on whether up to 10% lymphocytic inflammation was considered normal or abnormal. In another study, histopathological assessment also was used as a reference standard.^16^ The sensitivity and specificity of the Spec fPL test in that study were 67 and 91%, respectively, indicating, as in the previous study,^15^ that a positive result had a high probability of being a true positive but a negative result could not be used to rule out the diagnosis. This conclusion is similar to the conclusion drawn from our study, albeit using a different method of analysis.

In our study, abdominal ultrasonography was performed in all cats and changes consistent with pancreatitis were required to classify a cat as having definite or probable pancreatitis. Abdominal ultrasonography is both less expensive and less invasive than pancreatic biopsy. In 1 study, it was reported to have a sensitivity of 84% and a specificity of 75% for the diagnosis of pancreatitis in cats.^17^ However, in that study serum fPL was used as the standard to confirm pancreatitis, despite the fact that validation of the diagnostic utility of the fPL test is limited.^12^ In another study,^16^ in which histopathology was used to confirm the diagnosis of pancreatitis, the sensitivity of abdominal ultrasound examination was 67% and its specificity was 88%.

An additional complication is that the sensitivity and specificity of ultrasound imaging are highly dependent on the skill and experience of the ultrasonographer.^1^, ^3^, ^5^ Therefore, published results of studies performed at referral institutions may not be as relevant to ultrasonographic studies performed in general practice by nonspecialists. In addition, there is no standardized ultrasonographic grading scheme for pancreatitis in cats, and as a result, ultrasonographic interpretations can be very variable among clinicians.^18^, ^19^ Several studies have reported relatively low sensitivity of ultrasonographic diagnosis in cats with pancreatitis,^16^, ^20^, ^21^ indicating that a normal abdominal ultrasound examination does not rule out the diagnosis.

Assessing the sensitivity and specificity of the ultrasonographic diagnosis of pancreatitis was not the focus of our study. However, 36 cats in the possible pancreatitis group and 8 cats in the unlikely pancreatitis group had positive fPL results including several with very high results (more than twice the upper reference range). It therefore is feasible that some, if not many, of these cats did indeed have pancreatitis (either as their primary disease or a comorbidity) but negative ultrasonographic findings. Our results highlight the need to combine the Spec fPL test with abdominal ultrasound findings to help improve the diagnosis of pancreatitis in cats, because both tests are useful (but not infallible) in confirming the diagnosis if positive but neither test can rule out the diagnosis.

A secondary aim of our study was to review the clinical signs reported in cats suspected to have pancreatitis and to evaluate changes in selected biochemical tests in the diagnostic assessment of cats that may have pancreatitis. As Table 2 indicates, the most common clinical signs seen in all groups were lethargy, anorexia, vomiting, and weight loss, indicating that clinical findings are not reliable for differentiating pancreatitis from nonpancreatic disease.

Hypoalbuminemia, increased ALT and ALP activities, hyperbilirubinemia and hypocalcemia are reported as potential serum biochemical abnormalities in cats with pancreatitis.^1^, ^5^, ^11^, ^14^ Significant differences in serum albumin concentrations have been reported in cats with moderate to severe pancreatitis compared with healthy cats and cats with mild pancreatitis,^16^ a finding not replicated in our study of a much larger cohort of cats (n = 274 versus n = 29).^16^ Although significant differences in serum bilirubin concentrations were found among groups in our study, it was not diagnostically helpful because no significant difference in bilirubin concentrations was found between in cats in the definite or probable pancreatitis group and those in the unlikely pancreatitis group. No correlation was found between fPL and serum bilirubin concentration. No significant differences were found among groups in the other biochemical tests, a finding similar to a previous study.^16^

Our study had several limitations. It was a retrospective study conducted on a population of sick cats assessed and treated at a specialist referral hospital. The cases were a mixture of first opinion cases that entered through our first opinion emergency service and referral cases, but the proportion of each type of case could not be determined. Unfortunately, in only a small number of cats (n = 9) was pancreatitis confirmed visually, cytologically, or histopathologically (definite pancreatitis). However, a large group of cats (n = 58) had clinical signs and ultrasonographic changes consistent with pancreatitis (definite + probable pancreatitis group), and thus the diagnostic performance of fPL in this group of cats was clinically relevant.

In conclusion, our study supported the use of Spec fPL as part of the diagnostic evaluation of cats with suspected pancreatitis. However, our results must be interpreted with caution. A positive result increases the likelihood of the diagnosis, because in our study and others, the false‐positive rate appears to be low. It cannot, however, be used to rule out pancreatitis as a diagnosis because the false‐negative rate is relatively high. Approximately 25% of the cases classified as definite or probable pancreatitis in our study would have been missed if Spec fPL was the only diagnostic test used. Pancreatitis in cats remains a challenge to diagnose, and results from multiple diagnostic modalities should be assessed when making the diagnosis.

## 5 CONFLICT OF INTEREST DECLARATION

Authors declare no conflict of interest.

## 6 OFF‐LABEL ANTIMICROBIAL DECLARATION

Authors declare no off‐label use of antimicrobials. Institutional Animal Care and Use Committee (IACUC) or Other Approval Declaration. Authors declare no IACUC or other approval was needed.

## 7 HUMAN ETHICS APPROVAL DECLARATION

Authors declare human ethics approval was not needed for this study.
